# The Self-amplifying Inflammatory Cascade in Obstetric Antiphospholipid Syndrome: From Pathophysiology to Placenta-centric Immunotherapeutic Targets

**DOI:** 10.1007/s12016-026-09153-7

**Published:** 2026-03-31

**Authors:** Rui Yan, Yuxin Wei, Lu Chen, Shaodan Zhu, Xudong Dong

**Affiliations:** 1 Faculty of Life Science and Technology, Kunming University of Science and Technology, Kunming, 650500 People’s Republic of China; 2 The Obstetrical Department of the First People’s Hospital of Yunnan Province, The Affiliated Hospital of Kunming University of Science and Technology, Kunming, 650032 People’s Republic of China

**Keywords:** Obstetric Antiphospholipid Syndrome (OAPS), Inflammation, Placenta, Immunotherapy, Pathophysiology

## Abstract

Obstetric Antiphospholipid Syndrome (OAPS) is an autoimmune-driven pregnancy complication whose core pathology is a dysregulated inflammatory cascade at the maternal-fetal interface, rather than the traditionally emphasized thrombosis. Antiphospholipid antibodies (aPL) trigger a diverse array of innate immune pathways, including the complement system, neutrophils, and the inflammasome, leading to trophoblast dysfunction, poor placentation, and ultimately, adverse pregnancy outcomes such as recurrent miscarriage, preeclampsia, and fetal growth restriction. The current standard of care, low-dose aspirin combined with heparin, primarily targets coagulation and has limited efficacy in controlling the underlying inflammatory response, resulting in treatment failure in approximately 20–30% of patients. This review aims to redefine the inflammatory nature of OAPS, elaborate on the immune-pathological network centered on the placenta, and critically examine the pivotal role of the signaling cascade mediated by TLR4 that activates the downstream NF-κB transcription factor as a major regulatory node of the inflammatory response. In this context, experimental inhibitors target the initiation of this cascade at the receptor level to prevent the subsequent nuclear translocation of NF-κB. Building on this, we review the evolution of treatment strategies from traditional anticoagulants to immunosuppressants and propose that “targeting the local placental immune microenvironment” is the core direction for future therapies. Finally, this paper discusses a novel snail-inspired bioactive substance, specifically focusing on the natural glycosaminoglycan AFG (Achatina fulica glycosaminoglycan). This glycosaminoglycan is a non-anticoagulant heparin-like GAG purified from snail mucus, characterized by a regular repeating sequence of →4)-β-D-GlcNAc(1→4)-α-L-IdoA_2S_(1→. AFG functions as a specialized immunomodulator that stabilizes placental cellular signaling by precisely targeting the placental inflammatory axis.

## Introduction

### Beyond Thrombosis: A Paradigm Shift in OAPS Pathogenesis

Antiphospholipid Syndrome (APS) is an acquired, systemic, thrombo-inflammatory autoimmune disease characterized by the persistent presence of antiphospholipid antibodies (aPL) accompanied by vascular thrombosis and/or pregnancy morbidity [1]. For decades, APS has been primarily viewed as an acquired prothrombotic state. However, a growing body of evidence has prompted a profound reassessment of this traditional view, particularly within the realm of Obstetric Antiphospholipid Syndrome (OAPS). OAPS differs significantly from thrombotic APS in its clinical manifestations and pathological mechanisms; the latter is dominated by arterial and venous thrombotic events, whereas the former presents as a spectrum of pregnancy complications related to placental dysfunction [1].

The core evidence for this paradigm shift stems from histopathological studies of placentas from OAPS patients. Contrary to the expectation of widespread vascular thrombosis and infarction, the hallmark features of OAPS placentas are often inflammatory cell infiltration, complement deposition, trophoblast injury, defective spiral artery remodeling, and placental developmental defects, with thrombosis being neither a universal nor a primary pathological finding [2]. These observations strongly challenge the traditional model that posits placental ischemia and infarction as the sole etiology of OAPS. In its place, a new paradigm has emerged: OAPS is fundamentally a disease of “sterile inflammation” at the maternal-fetal interface, driven by aPL-mediated dysregulation of the innate immune system. This shifting paradigm highlights that both established and newly identified pathogenic mechanisms converge on sterile inflammation at the maternal-fetal interface [3, 4].

### Clinical Burden and Unmet Needs: Limitations of Current Anticoagulation-Centered Therapy

OAPS imposes a significant burden on maternal and infant health, with clinical manifestations including recurrent early miscarriages, mid-to-late-term fetal demise, severe preeclampsia (PE), and intrauterine growth restriction (IUGR) [5]. Currently, the standard of care (SOC) for OAPS is low-dose aspirin (LDA) combined with prophylactic or therapeutic doses of heparin [6]. Although this regimen has significantly improved pregnancy outcomes, its success is far from absolute. Clinical data show that up to 20–30% of OAPS patients experience adverse pregnancy outcomes despite receiving standard anticoagulant therapy, representing a substantial treatment gap [7].

This persistent treatment failure rate does not arise from the ineffectiveness of anticoagulant drugs per se, but rather from a mismatch between the therapeutic strategy and the core pathological mechanism. While heparin possesses certain anti-inflammatory and complement-inhibiting “pleiotropic” effects beyond its anticoagulant action [8], these effects are secondary and often insufficient to completely abrogate the placental pathological cascade ignited by aPL. This is particularly true for late-pregnancy complications, such as severe PE and IUGR, which are closely linked to chronic inflammation and placental insufficiency, where purely anticoagulant therapy is often inadequate [9]. Therefore, the redefinition of OAPS is not merely an academic revision but a pressing clinical necessity. It directly points to the development of novel, non-anticoagulant therapies targeting inflammatory pathways as the key to resolving the current clinical dilemma.

## The Placenta: The Immunological Battlefield of OAPS

In OAPS, the placenta is not only the hub for maternal-fetal exchange but also the primary battlefield where the immune system is dysregulated under the assault of aPL. As the initiating factor, aPL triggers a series of interconnected and self-amplifying inflammatory reactions at the maternal-fetal interface, ultimately leading to impaired placental function.

### Antiphospholipid Antibodies: The Initiators of Placental Injury

**Fig. 1:**
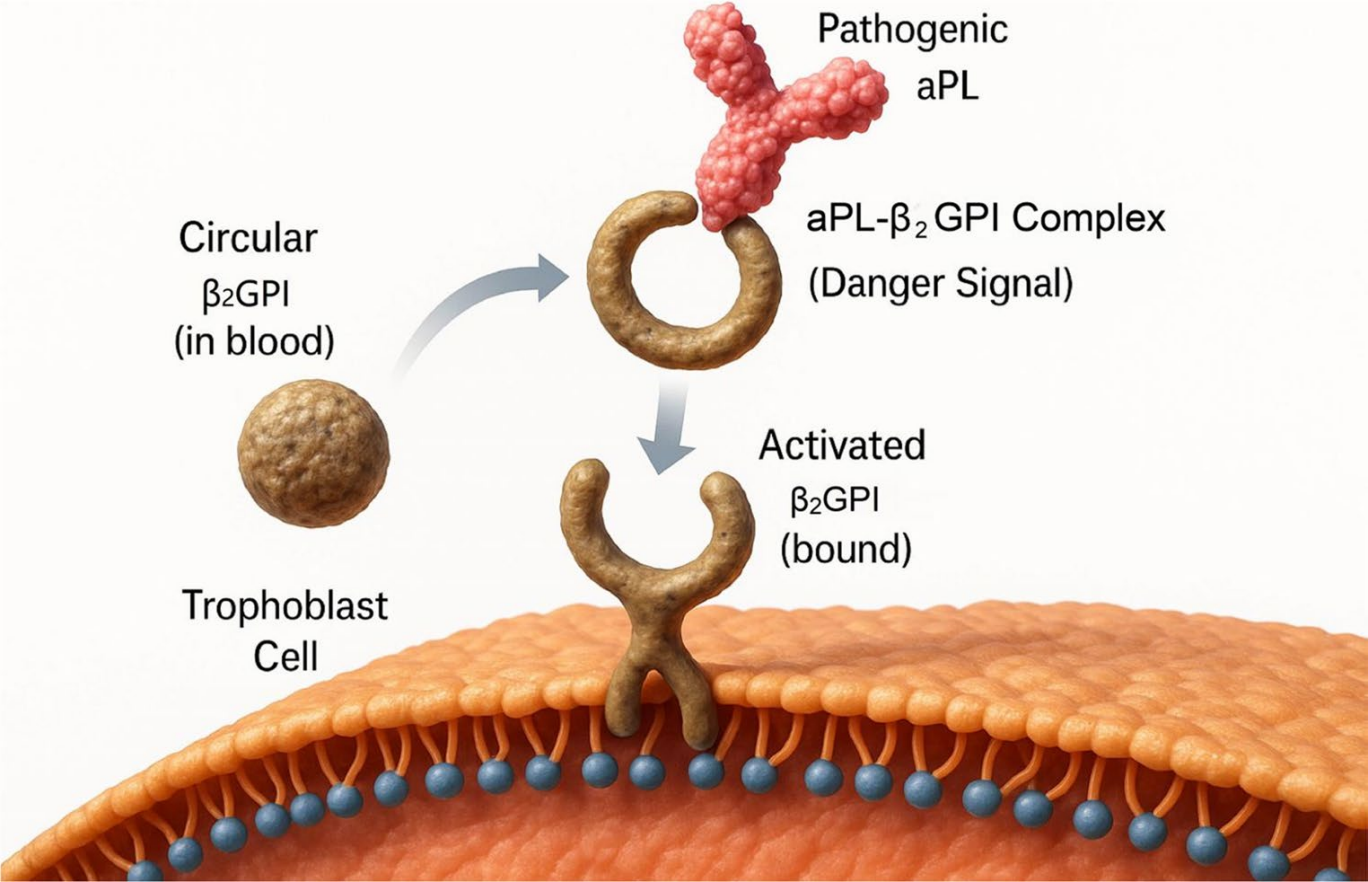
The “First Hit” Mechanism of Placental Injury in Obstetric Antiphospholipid Syndrome (OAPS). In the maternal circulation, β2-glycoprotein I (β2GPI) exists in a circular conformation. Upon binding to anionic phospholipids on the surface of placental trophoblast cells, β2GPI undergoes a critical conformational change, exposing a cryptic epitope. Pathogenic antiphospholipid antibodies (aPL) then bind to this activated form of β2GPI, creating a stable, multivalent aPL-β2GPI complex. This complex functions as a potent danger signal, anchoring to the cell surface and triggering the downstream inflammatory cascade characteristic of OAPS

The primary targets of pathogenic aPL are not phospholipids themselves, but rather plasma proteins that bind to anionic phospholipids on cell membranes, the most critical of which is β2-glycoprotein I (β2GPI) [10]. In a normal physiological state,

β2GPI exists in a circular conformation. However, when it binds to anionic phospholipids on the surface of placental trophoblast cells, it undergoes a conformational change, exposing a cryptic epitope. Pathogenic aPL recognize and bind to this neoepitope, forming an aPL-β2GPI complex that acts as a “danger signal,” anchoring to the trophoblast surface and constituting the “first hit” that initiates the subsequent pathological cascade [11] (Fig. 1).

### The Inflammatory Cascade at the Maternal-Fetal Interface

Once formed, the aPL-β2GPI complex recruits and activates multiple innate immune effector mechanisms, forming an interconnected and self-amplifying pathological network that constitutes the deregulated inflammatory cascade of OAPS.

#### The Complement System: A Core Mediator of Tissue Damage

The binding of aPL to their targets on the trophoblast surface effectively activates the classical complement pathway [12]. This process leads to the abundant deposition of complement fragments C4d and C3b on the trophoblast cell membrane, which has become one of the hallmark histological findings in placentas of OAPS patients [13]. The complement cascade ultimately generates two key effector molecules: the potent anaphylatoxins C3a and C5a, and the terminal product, the membrane attack complex (MAC, C5b-9) [14].

Among these, C5a acts as a powerful chemoattractant, recruiting inflammatory cells such as neutrophils to the local placental environment, thereby amplifying the inflammatory response. Concurrently, both C5a and MAC can directly injure trophoblasts and vascular endothelial cells, inducing them to express pro-inflammatory and pro-coagulant molecules, thus exacerbating tissue damage [15]. Animal models provide decisive evidence for the central role of complement in OAPS: in aPL-induced miscarriage models, mice deficient in C3 or C5 are completely resistant to the pathogenic effects of aPL, confirming that complement activation is a necessary step in mediating placental injury and embryo loss [16].

#### Neutrophils and NETosis: Weaving the Inflammatory Web

Neutrophils are key cellular effectors in the OAPS pathological cascade. aPL can directly activate neutrophils via β2GPI, promoting their massive infiltration into the decidua and placental intervillous space [17]. Activated neutrophils undergo a unique form of cell death known as NETosis, forming Neutrophil Extracellular Traps (NETs)—web-like structures composed of decondensed chromatin, histones, and granular proteins like myeloperoxidase (MPO) [18].

NETs play multiple destructive roles in the pathogenesis of OAPS. First, they provide a physical scaffold for platelet and coagulation factor aggregation, directly promoting thrombosis. Second, NET components (such as histones) are directly cytotoxic to trophoblasts and endothelial cells. Furthermore, as potent endogenous danger signals, NETs can further activate other immune pathways, such as the complement system [19]. Recent research has further revealed that NETs can exacerbate placental injury by inducing trophoblast apoptosis and activating BNIP3-mediated mitophagy, providing a new molecular mechanism for the pathogenic role of NETs in OAPS [20].

#### The Inflammasome: The Intracellular Engine of IL-1β Production

The NLRP3 inflammasome is a multiprotein complex within the cytoplasm that serves as a key platform for sensing intracellular danger signals and initiating a potent inflammatory response. Its activation leads to the cleavage and activation of interleukin-1β (IL-1β) and IL-18, two cytokines that are powerful amplifiers of inflammation [21].

In the trophoblasts of OAPS, NLRP3 inflammasome activation follows the classic “two-signal” model. aPL, by binding to Toll-like receptor 4 (TLR4), provides “signal one,” inducing the transcription and synthesis of intracellular pro-IL-1β (the precursor to IL-1β) [22]. Subsequently, endogenous damage−associated molecular patterns (DAMPs), such as uric acid, generated by the aPL-TLR4 signaling pathway itself, act as “signal two,” triggering the assembly of the NLRP3 inflammasome and the activation of caspase − 1. This ultimately cleaves pro − IL−1β into its mature, biologically active form, which is then released from the cell [23, 24]. Notably, this inflammasome activation is not restricted to the trophoblast but is also robustly expressed in the human endometrium, representing a crucial pathological event on the “maternal side” of the implantation site [25].

Crucially, these pathways function synergistically rather than in isolation. Complement-derived C5a recruits neutrophils, while NETs provide a scaffold for further complement activation. Concurrently, cellular stress induced by these effectors releases DAMPs, triggering the NLRP3 inflammasome and IL-1β release, which further propagates neutrophil recruitment.

Trophoblast Dysfunction: The Cellular Consequence of Inflammatory Attack.

The pro-inflammatory signaling initiated by the aPL-TLR4 axis, and its subsequent recruitment of innate immune effectors, impairs the critical physiological functions of trophoblast cells, bridging the gap between molecular pathology and clinical phenotype. Extensive research has confirmed that aPL can:

Inhibit trophoblast viability, proliferation, and syncytialization: The syncytiotrophoblast is a crucial structure for maintaining the placental barrier and hormone secretion. Its impaired formation severely compromises placental function [26].

Hinder trophoblast migration and invasion: The invasion of trophoblasts into the maternal decidua is essential for the successful remodeling of spiral arteries. aPL inhibit this process by downregulating key molecules like Matrix Metalloproteinase-1 (MMP1), leading to shallow placentation and insufficient utero-placental blood perfusion [27]. Furthermore, aPL significantly impairs human endometrial angiogenesis and endothelial cell function, representing a critical defect on the ‘maternal side’ of the interface [28–31].

Induce trophoblast cell death: aPL can induce both apoptosis and pyroptosis in trophoblasts. The latter is a highly pro-inflammatory form of cell death that releases a large number of inflammatory mediators, further exacerbating local inflammation [32]. The integration of these inflammation-mediated cellular dysfunctions ultimately culminates in the macroscopic pathological outcomes of poor placental development, directly leading to the various adverse pregnancy outcomes associated with OAPS (Table 1).

**Table 1:** Key inflammatory pathways and mediators in OAPS pathogenesis

Pathway	Activation Trigger	Key Mediators	Downstream Effects on Placenta	References
Complement System	aPL-β2GPI complex binding to trophoblasts	C3b, C4d, C5a, C5b-9 (MAC)	Trophoblast injury, inflammation, neutrophil recruitment, procoagulant state	[63]
Neutrophils & NETosis	Direct aPL activation, C5a chemoattraction	NETs (DNA, histones, MPO)	Direct cytotoxicity, thrombotic scaffold, source of autoantigens, induction of mitophagy/apoptosis	[64]
Inflammasome	aPL-TLR4 binding (Signal 1) + DAMPs (Signal 2)	NLRP3, ASC, Caspase-1, IL-1β	Potent local inflammation, cellular pyroptosis	[65]

## TLR4-mediated NF-κB activation cascade: A Major Regulatory Node of Placental Inflammation

Within the complex inflammatory network of OAPS, the Toll-like receptor 4 (TLR4) and its downstream Nuclear Factor-κB(NF − κB) signaling pathway occupy a central hub position, acting as the “pivotal regulatory node” that translates the extracellular aPL signal into an intracellular program of inflammatory gene expression.

### TLR4: The Key Receptor for aPL-Mediated Cellular Activation

Although the cell membrane protein Annexin A2 is considered a high-affinity binding receptor for β2GPI, it lacks a transmembrane structure and an intracellular signaling domain, making it incapable of directly initiating intracellular responses. Substantial evidence indicates that the transmembrane protein TLR4 acts as its “co-receptor” or “adapter,” playing an indispensable role in the transmembrane transduction of the aPL signal [33]. The activating effects of aPL on human trophoblasts and endothelial cells have been demonstrated to involve the TLR4/MD2/CD14 complex, as evidenced by studies using TLR4-deficient models or specific inhibitors that abolish aPL-induced pro-inflammatory responses [34]. Although the precise dependency and the role of co-receptors such as Annexin A2 are still being elucidated, current evidence suggests that TLR4 serves as a critical transducer of aPL-induced inflammatory signals in the placental microenvironment. This discovery was a landmark, as it was the first to directly link a classic innate immune receptor, typically used to sense pathogens, to the pathological process of an autoimmune disease.

### Downstream Signaling: NF-κB and the Transcriptional Amplification of Inflammation

**Fig. 2:**
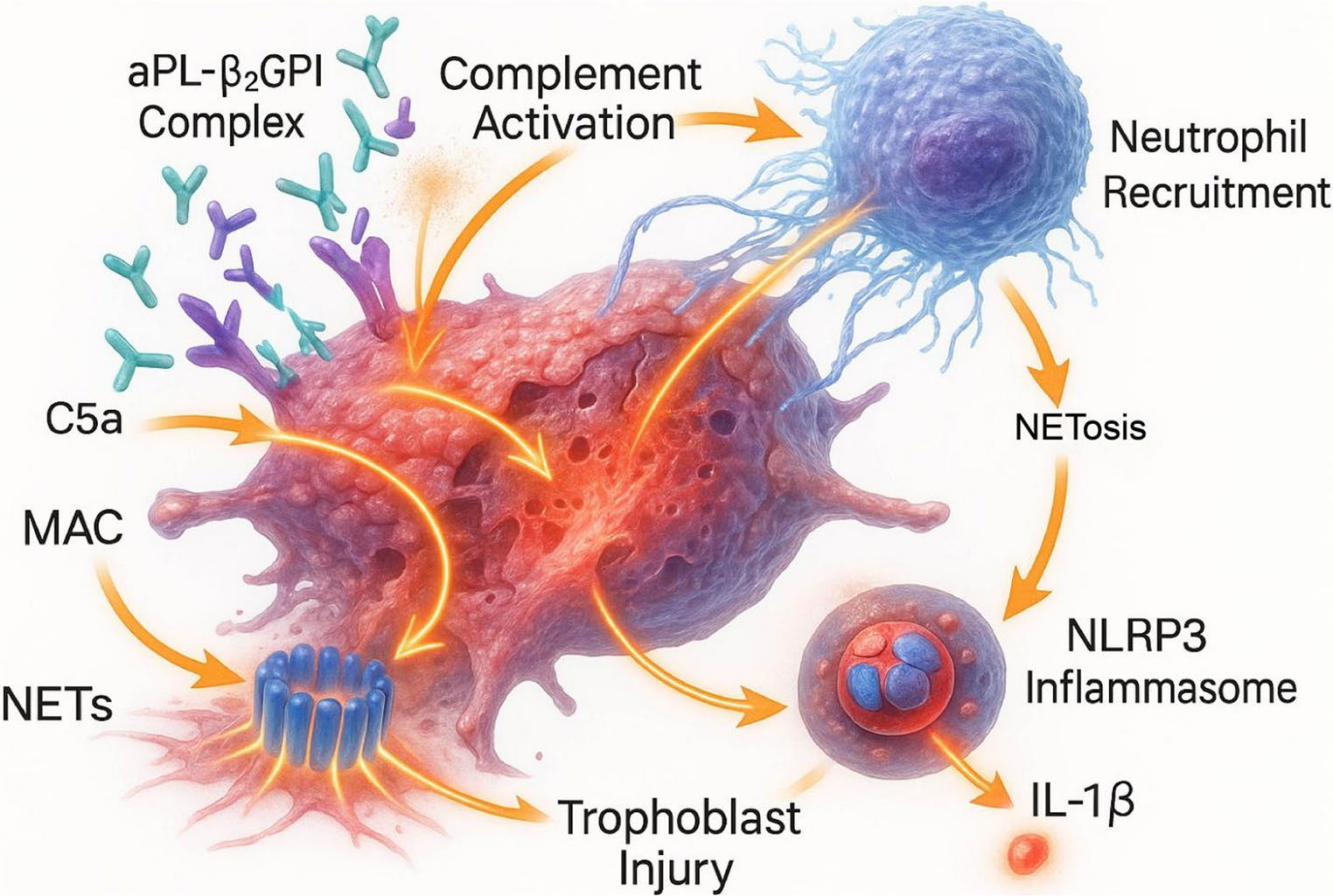
The Interconnected Inflammatory Cascade Constituting the Placental “Inflammatory Storm” in OAPS. The aPL-β2GPI complex triggers a self-amplifying pathological triad of innate immune pathways. (1) Complement Activation: The complex activates the classical complement pathway, generating anaphylatoxin C5a and the membrane attack complex (MAC). C5a recruits neutrophils, while MAC directly injures the trophoblast. (2) Neutrophil Recruitment and NETosis: Neutrophils recruited to the placenta undergo NETosis, releasing Neutrophil Extracellular Traps (NETs) that are directly cytotoxic and prothrombotic. (3) NLRP3 Inflammasome Activation: Cellular stress and damage signals activate the NLRP3 inflammasome within the trophoblast, leading to the maturation and release of the potent pro-inflammatory cytokine IL-1β. These pathways create a vicious cycle, amplifying inflammation and culminating in severe trophoblast injury

The activation of TLR4 initiates a classic downstream signaling cascade that ultimately converges on the activation of the “principal orchestrator” of inflammatory transcription factors—NF-κB. In a resting state, NF-κB is sequestered in the cytoplasm by the inhibitory protein IκB. The TLR4 signaling pathway phosphorylates and degrades IκB, liberating NF-κB to translocate into the nucleus. Once in the nucleus, NF-κB binds to the promoter regions of numerous pro-inflammatory genes, initiating the transcription of a vast array of genes, including cytokines (e.g., TNF-α, IL-6), chemokines, and adhesion molecules [35]. This transcriptional program is the molecular engine that amplifies and sustains the inflammatory response at the maternal-fetal interface, transforming the initial aPL binding event into a vicious inflammatory cycle (Fig. 2).

### The TLR4/NF-κB Axis as a Prime Therapeutic Target: Evidence and Rationale

The upstream, central position of the TLR4/NF-κB signaling axis in the OAPS inflammatory cascade makes it a highly attractive therapeutic target. Compared to targeting a single downstream effector molecule (such as complement C5 or a specific cytokine), inhibiting this “pivotal regulatory node” can more comprehensively and effectively block the initiation and amplification of the entire inflammatory network. By targeting this central node, experimental glycosaminoglycans targeting the TLR4-mediated pathway can potentially suppress the production of pro-inflammatory cytokines and attenuate the ‘priming’ signal required for NLRP3 inflammasome activation.

The therapeutic potential of AFG in OAPS is supported by its proven immunomodulatory performance in other sterile inflammatory models. Given that AFG significantly suppresses the TLR4/NF-κB signaling axis and promotes anti-inflammatory macrophage polarization in wound healing and diabetic complications [36, 37], it is highly plausible that this glycosaminoglycan could similarly target the placental inflammatory hub. We hypothesize that by correcting the “erroneous signal interpretation” mediated by TLR4, AFG could potentially restore immune homeostasis at the maternal-fetal interface—a strategy that warrants rigorous validation in OAPS-specific animal models. This provides the most direct evidence for the TLR4/NF − κB axis as a key and druggable therapeutic target in OAPS. This mechanistic elucidation also fundamentally reclassifies OAPS as an innate immunity-driven autoimmune disease. Pathogenic autoantibodies (aPL) hijack the body’s ancient pathogen defense pathway (TLR4), mistakenly directing it to attack self-tissue (the placenta), which explains the rapid and fierce nature of the OAPS inflammatory response. Therefore, therapies like AFG, by correcting this “erroneous signal interpretation,” hold the promise of precisely restoring immune homeostasis at the maternal-fetal interface without causing widespread immunosuppression.

While the TLR4/NF-κB axis acts as a critical driver of placental inflammation, it is essential to acknowledge that OAPS pathogenesis is highly multifactorial, involving a distinct thromboinflammatory signature characterized by broad complement and neutrophil activation [38]. Consequently, this pathway does not act in isolation but operates alongside parallel mechanisms, such as signaling via intracellular TLR7/8 [39], apolipoprotein E receptor 2 (ApoER2), and direct complement-mediated damage. Thus, TLR4 represents a prominent component of a broader mechanistic network rather than an exclusive causal dominant.

## The Evolution of OAPS Treatment Strategies

The history of OAPS treatment reflects our deepening understanding of its pathological mechanisms. From an initial focus solely on anticoagulation, to attempts at broad-spectrum immunosuppression, and now to the exploration of more targeted immunomodulation, the trajectory of treatment strategies clearly points toward inflammation as the core pathology.

### Standard of Care: Low-Dose Aspirin and Heparin

#### Pleiotropic Mechanisms Beyond Anticoagulation

Low-dose aspirin (LDA) combined with prophylactic or therapeutic doses of heparin (unfractionated or low-molecular-weight) is the currently accepted first-line standard of care. Their classic mechanisms of action are, respectively: LDA inhibits platelet cyclooxygenase to block thromboxane A2 synthesis, thereby exerting an antiplatelet effect; heparin enhances the activity of antithrombin III to exert its anticoagulant effect [40].

However, their partial efficacy in OAPS may also be attributed to their “pleiotropic” effects beyond anticoagulation. Studies have shown that heparin can inhibit the activation of the complement system and, to some extent, prevent the binding of aPL to trophoblast cells [41]. Significantly, low-molecular-weight heparins (LMWHs) have also been shown to directly counteract aPL-mediated inhibition of endometrial angiogenesis and prevent trophoblast apoptosis, providing protective mechanisms entirely independent of their anticoagulant properties [42–44].

Furthermore, LDA exerts distinct anti-inflammatory effects by modulating local placental cytokine profiles and preventing vascular inflammation through the inhibition of the NF-κB pathway [45].

#### Efficacy, Safety, and Persistent Treatment Failures

The LDA and heparin combination has significantly increased the live birth rate in OAPS patients, with success rates reaching 70–80% in preventing recurrent early miscarriages [46]. However, the limitations of this regimen are equally prominent. It is less effective in preventing late-pregnancy complications that are more closely associated with chronic placental inflammation and insufficiency, such as severe preeclampsia, IUGR, and stillbirth [47]. This again corroborates the central thesis: anticoagulation is an incomplete treatment strategy that fails to eradicate the root cause of inflammation.

### Adjunctive Immunomodulatory Therapies for Refractory OAPS

For the 20–30% of patients with refractory OAPS who fail standard treatment, the addition of immunomodulatory drugs is considered.

#### Glucocorticoids: A Historical Perspective and Cautious Application

Historically, high-dose prednisone was used to treat OAPS due to its potent immunosuppressive effects [48]. However, subsequent studies found that, compared to heparin, high-dose steroids offered no clear additional benefit and significantly increased the risk of maternal and fetal complications such as gestational diabetes, hypertension, and preterm birth. Consequently, their use has been largely abandoned [49]. Currently, only in a very small, highly selected group of refractory cases might short-term use of low-dose prednisone (e.g., 10 mg/day) be considered in early pregnancy, but its efficacy remains controversial, and the risks must be carefully weighed [50].

#### Hydroxychloroquine: A Promising Pleiotropic Agent

Hydroxychloroquine (HCQ) is increasingly becoming an important adjunctive therapy in the treatment of refractory OAPS [51]. Its efficacy stems from its multiple immunomodulatory effects that target the pathological mechanisms of OAPS:

Inhibition of TLR signaling pathways (including TLR4 and intracellular TLR7/9) directly intervenes in the initial stages of inflammation [52]. Inhibition of complement activation. Mitigation of NET-induced trophoblast pyroptosis by inhibiting the MAPK and NF-κB signaling pathways [53]. Antithrombotic effects through the inhibition of platelet activation [54].

Several retrospective studies and meta-analyses have shown that adding HCQ to the standard of care is associated with higher live birth rates and lower complication rates, making it a very promising treatment option for high-risk OAPS patients [51]. This evolution in treatment—from using broad-spectrum immunosuppressants (steroids) to multi-modal immunomodulators (HCQ)—Paves the way for the development of highly specific targeted therapies (such as AFG) and demonstrates a trend toward therapies with better risk-benefit profiles through increased specificity.

#### Intravenous Immunoglobulin (IVIg) Therapy

For severe or highly refractory OAPS cases that fail to respond to standard anticoagulation and first-line immunomodulators, high-dose intravenous immunoglobulin (IVIg) has emerged as a valuable rescue option. IVIg exerts broad immunomodulatory effects, including the neutralization of pathogenic autoantibodies, inhibition of complement activation, and modulation of cytokine networks. Recent clinical studies have demonstrated that IVIg can significantly improve pregnancy outcomes and live birth rates in women with refractory OAPS [55].

## The Future of OAPS Therapy: Precision Targeting of the Placental Immune Microenvironment

As our understanding of the pathological mechanisms of OAPS deepens, future treatment strategies are moving towards greater precision and safety, with the core concept being “targeting the local placental immune microenvironment.”

### The Rationale for Placenta-Centric Therapy

The pathological damage in OAPS is primarily confined to the placenta; therefore, an ideal treatment should also be localized to this site as much as possible. Systemic immunosuppressants or biologic agents inevitably carry risks such as decreased maternal immunity, increased risk of infection, and potential fetal exposure [56]. A placenta-centric therapeutic strategy aims to deliver drugs precisely to the site of pathology, thereby maximizing local efficacy while minimizing systemic exposure and off-target effects on both the mother and fetus, achieving a unity of efficacy and safety [57].

### Emerging Biologic Agents: Lessons and Insights from Systemic Therapies

In recent years, some potent biologic agents have shown promise in the treatment of severe or catastrophic APS, providing clinical evidence for the importance of key pathways, but their application in OAPS still faces challenges.

#### Targeting Complement: The Promise and Challenges of C5 Inhibitors

Eculizumab, a monoclonal antibody targeting complement C5, has demonstrated a powerful ability to rapidly control thrombotic storms in several case reports of refractory and catastrophic APS [58]. This provides strong clinical proof-of-concept for the critical role of complement in APS pathogenesis. However, its routine use in OAPS is limited by its systemic effects, high cost, and the need for continuous administration. For a localized disease, it remains an overly potent and systemically distributed agent.

#### Other Systemic Therapies: Cytokine Blockade and B-Cell Depletion

TNF-α inhibitors (such as adalimumab) and B-cell depleting drugs (such as rituximab) have also been used to treat severe, refractory APS [59]. Although they act on relevant pathological pathways, their use during pregnancy is particularly complex, requiring careful assessment of their impact on fetal immune system development and maternal infection risk [60]. Typically, these therapies are reserved for extreme situations where the mother’s health is under significant threat.

### AFG: A Novel Snail-inspired Glycosaminoglycan Targeting the Placental Inflammatory Axis

**Fig. 3:**
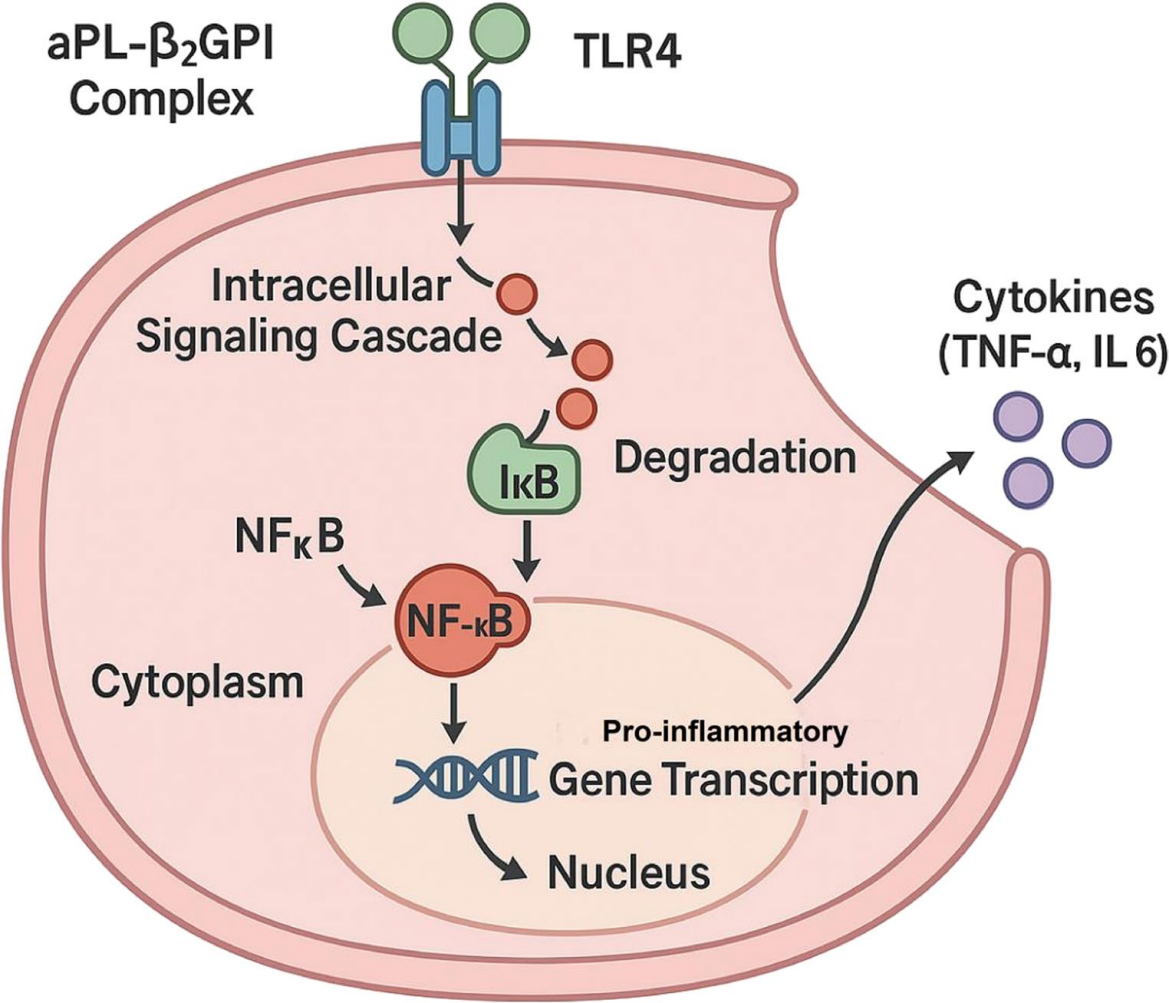
The TLR4/NF-κB Signaling Axis as the “Master Switch” of Placental Inflammation in OAPS. The binding of the aPL-β2GPI complex to the Toll-like receptor 4 (TLR4) on the trophoblast surface initiates an intracellular signaling cascade. In the cytoplasm, this cascade leads to the phosphorylation and subsequent degradation of the inhibitory protein IκB. The degradation of IκB liberates the transcription factor Nuclear Factor-κB (NF-κB), allowing it to translocate from the cytoplasm into the nucleus. Within the nucleus, NF-κB binds to the promoter regions of target genes, driving the pro-inflammatory gene transcription of numerous cytokines (e.g., TNF-α, IL-6), chemokines, and adhesion molecules that fuel the placental inflammatory storm

The experimental compound Achatina fulica glycosaminoglycan (AFG) is a research-stage, non-anticoagulant heparin-like natural polymer purified from snail mucus. Structurally characterized by a regular repeating sequence of →4)-β-D-GlcNAc(1→4)-α-L-IdoA2S(1→, AFG exhibits significant anti-inflammatory efficacy in preliminary preclinical models. The experimental therapeutic potential of AFG is characterized by its preliminary capacity to modulate the TLR4-mediated signaling axis, promote the transition of macrophages toward an anti-inflammatory phenotype, and maintain a non-anticoagulant profile, which avoids the bleeding complications associated with heparin **(**Fig. 3).

Crucially, any discussion of AFG’s therapeutic potential must be strictly contextualized within its current preclinical developmental stage. At present, there is a distinct absence of OAPS-specific in vivo validation. Furthermore, critical data regarding its safety profile during pregnancy, potential teratogenicity, and transplacental transfer rates are currently unavailable. Therefore, extrapolations of its efficacy to human OAPS remain hypothesis-driven and require rigorous future experimental validation.

### Technological Frontier: Nanoparticle-Mediated Placenta-Targeted Drug Delivery

To maximize the potential of precision drugs like AFG, they can be combined with cutting-edge drug delivery technologies. In recent years, the concept of “placenta-anchored” drug delivery systems has gained significant attention [61]. These systems use nanoparticles as carriers, modified on their surface with ligands that can specifically recognize molecules on the placental surface (such as peptides targeting placental chondroitin sulfate A). This allows the drug to “dock” and accumulate in the placental tissue after traversing the maternal circulation, achieving a high local concentration while significantly reducing the risk of the drug entering the fetal circulation [62].

**Fig. 4:**
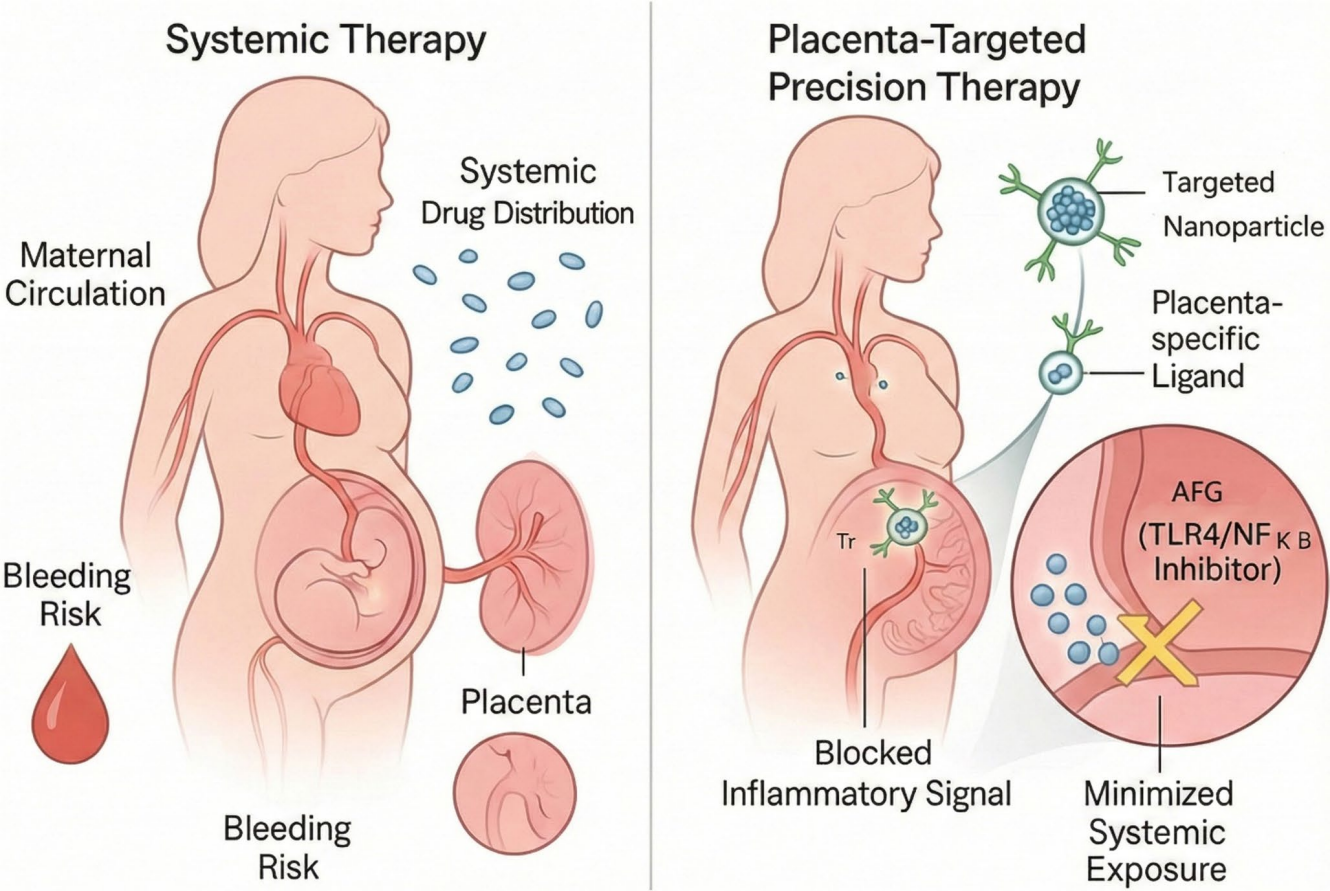
Comparison of Therapeutic Strategies for OAPS: From Systemic Treatment to Placenta-Targeted Precision Therapy. (Left Panel) Systemic Therapy: The current standard of care, such as heparin and aspirin, is distributed throughout the maternal circulation. This systemic drug distribution carries an inherent bleeding risk and fails to specifically target the placental inflammation, leading to treatment failures. (Right Panel) Placenta-Targeted Precision Therapy: A future therapeutic model involving a targeted nanoparticle encapsulating a novel inhibitor, *Achatina fulica* glycosaminoglycan (AFG). The nanoparticle is engineered with placenta-specific ligands, enabling it to accumulate at the site of pathology. AFG is then released locally to specifically block the inflammatory signal (e.g., the TLR4/NF-κB pathway), thereby maximizing therapeutic efficacy while minimizing systemic exposure and associated side effects for both the mother and fetus

It is foreseeable that loading an experimental glycosaminoglycan like AFG into a placenta-targeted nano-delivery system will constitute the targeted intervention model for OAPS: delivering the right drug, to the right place, at the right time. This strategy holds the promise of achieving an unprecedented unity of efficacy and safety, bringing true hope to patients with OAPS (Table 2 and Fig. 4).

**Table 2:** Comparative analysis of treatment strategies for obstetric antiphospholipid syndrome

Treatment Strategy	Primary Target	Mechanism of Action	Site of Action	Key Advantages	Key Disadvantages/Limitations
Standard of Care (LDA + Heparin) [66]	Platelets/Coagulation Factors	Antiplatelet/Anticoagulant	Systemic	Effective for early miscarriage (~ 75% live birth rate)	20–30% failure rate, bleeding risk, poor efficacy for late complications
Glucocorticoids [67]	Broad Immunosuppression	Anti-inflammatory/Immunosuppressive	Systemic	Potent immunosuppression	Significant maternal and fetal side effects
Hydroxychloroquine (HCQ) [68]	Multiple Targets (TLRs, complement, etc.)	Multi-modal immunomodulation	Systemic	Good safety profile, acts on multiple pathological pathways	Adjunctive therapy, RCT evidence pending
Complement Inhibitors (e.g., Eculizumab) [58]	Complement C5	Inhibits the terminal complement pathway	Systemic	Powerful effect in catastrophic APS	Systemic immunosuppression, high cost, limited OAPS data
AFG (Snail-inspired Glycosaminoglycan) [37]	TLR4/NF-κB Axis and Macrophage Polarization	Precision inhibition of the TLR4/NF-κB signaling axis and promotion of the M2 macrophage transition to restore placental immune homeostasis	Placenta-centric	Non-anticoagulant, targets the root cause, and promotes tissue regeneration	Currently in the preclinical research stage; direct efficacy and long-term safety in the human placental microenvironment require further validation

## Conclusion and Future Perspectives

In summary, the core pathophysiology of Obstetric Antiphospholipid Syndrome (OAPS) is a pathological cascade driven by the innate immune system at the maternal-fetal interface, rather than simple thrombosis. A misinterpretation of the disease’s fundamental nature is the root cause of the 20–30% failure rate of the current anticoagulation-centered treatment strategy.

Future therapeutic breakthroughs will inevitably require a shift from “anti-coagulation” to “anti-inflammation,” from “systemic” to “local,” and from “broad-spectrum” to “precision.” This review has systematically elucidated how aPL ignite a destructive deregulated inflammatory cascade in the placenta by activating pathways involving complement, neutrophils, and the inflammasome. Crucially, we have identified the TLR4/NF-κB signaling axis as the “pivotal regulatory node” of this cascade, the central hub connecting the autoantibody to the downstream inflammatory effectors.

Therefore, placenta-centric immunotherapies that precisely target key inflammatory nodes represent the future direction of OAPS treatment. Novel glycosaminoglycan-based inhibitors, exemplified by AFG, offer a highly promising candidate solution by specifically blocking the TLR4/NF-κB axis. Combined with nano-targeted delivery technologies, we are poised to develop a potential therapeutic approach that can deliver drugs to the site of pathology with high efficiency and safety. Nevertheless, it is critical to recognize that AFG is currently in the preclinical research stage. Its progression toward clinical implementation faces several primary technical bottlenecks that must be systematically addressed. These include the rigorous validation of long-term immunological safety within the human placental microenvironment, the optimization of penetration efficiency to ensure therapeutic molecules effectively reach target cells across the complex placental barrier, and the establishment of protocols for the large-scale standardization of AFG production to ensure consistent purity and biological activity. Future research should focus on validating the efficacy and safety of these novel targeted strategies through well-designed clinical trials, ultimately aiming to change the treatment landscape of OAPS and bring new hope to the patients who suffer from it.

## Data Availability

No datasets were generated or analysed during the current study.
